# Grass pollen allergoids conjugated with mannan for subcutaneous and sublingual immunotherapy: a dose-finding study

**DOI:** 10.3389/fimmu.2024.1431351

**Published:** 2024-06-26

**Authors:** Pedro Ojeda, María Concepción Barjau, Javier Subiza, Antonio Moreno, Isabel Ojeda, Emilio Solano, Alicia Alonso, Raquel Caballero, Sandra Del Pozo, Marta Gómez-Perosanz, José Luis Sánchez-Trincado, Cristina Benito-Villalvilla, Alba Angelina, Irene Soria, Pedro A. Reche, Oscar Palomares, José Luis Subiza, Miguel Casanovas

**Affiliations:** ^1^ Clínica de Asma y Alergia Dres. Ojeda, Madrid, Spain; ^2^ Centro de Asma y Alergia Subiza, Madrid, Spain; ^3^ Clínica Atlas, Aranjuez, Spain; ^4^ Servicio de Alergia, Hospital Universitario Ramón y Cajal, Madrid, Spain; ^5^ Clínica Alianza Médica, Valladolid, Spain; ^6^ Inmunotek, Alcalá de Henares, Spain; ^7^ Department of Medicine and Medical Specialities, Faculty of Medicine, University of Alcalá de Henares, Alcalá de Henares, Spain; ^8^ Department of Immunology & O2, School of Medicine, University Complutense of Madrid, Madrid, Spain; ^9^ Department of Biochemistry and Molecular Biology, School of Chemistry, University Complutense of Madrid, Madrid, Spain

**Keywords:** clinical trial, polymerized, allergoid, mannan, immunotherapy, grass pollen

## Abstract

**Background:**

Polymerized allergoids conjugated with mannan represent a novel approach of allergen immunotherapy targeting dendritic cells. In this study, we aimed to determine the optimal dose of mannan-allergoid conjugates derived from grass pollen (*Phleum pratense* and *Dactylis glomerata*) administered via either the subcutaneous or sublingual route.

**Methods:**

A randomized, double-blind, placebo-controlled trial with a double-dummy design was conducted, involving 162 participants across 12 centers in Spain. Subjects were randomly allocated to one of nine different treatment groups, each receiving either placebo or active treatment at doses of 500, 1,000, 3,000, or 5,000 mTU/mL over four months. Each participant received five subcutaneous (SC) doses of 0.5 mL each, every 30 days, and a daily sublingual (SL) dose of 0.2 mL. Participants who received active treatment through SC, received placebo through SL. Participants who received active treatment through SL, received placebo SC. One Group, as control, received bot SC and SL placebo. The primary efficacy outcome was the improvement in titrated nasal provocation tests (NPT) at the end of the study compared to baseline. Secondary outcomes included specific antibody (IgG4, IgE) and cellular (IL-10 producing and regulatory T cell) responses. All adverse events and side reactions were recorded and assessed.

**Results:**

Post-treatment, the active groups showed improvements in NPT ranging from 33% to 53%, with the highest doses showing the greatest improvements regardless of the administration route. In comparison, the placebo group showed a 12% improvement. Significant differences over placebo were observed at doses of 3,000 mTU/mL (p=0.049 for SL, p=0.015 for SC) and 5,000 mTU/mL (p=0.011 for SL, p=0.015 for SC). A dose-dependent increase in IgG4 was observed following SC administration, and an increase in IL-10 producing cells for both routes of administration. No serious systemic or local adverse reactions were recorded, and no adrenaline was required.

**Conclusion:**

Grass pollen immunotherapy with mannan-allergoid conjugates was found to be safe and efficacious in achieving the primary outcome, whether administered via the subcutaneous or sublingual routes, at doses of 3,000 and 5,000 mTU/mL.

**Clinical trial registration:**

https://www.clinicaltrialsregister.eu/ctr-search (EudraCT), identifier 2014–005471–88; https://www.clinicaltrials.gov, identifier NCT02654223.

## 1 Introduction

Grass pollen is a major cause of seasonal allergies in temperate climates, affecting up to 30% of the population in Western countries (1, 2). Since the pioneering work of Noon & Freeman (3), grass pollen-specific immunotherapy is recognized as the sole etiological treatment. Unlike symptomatic treatments, allergen immunotherapy (AIT) offers long lasting effects by inducing long-term tolerance to allergens (4). However, the requirement for prolonged courses, typically 3 to 5 years of treatment, poses a challenge to patient compliance (5, 6).

It is widely acknowledged that successful AIT often requires the administration of relatively high doses of allergens, which are likely to induce allergen-specific antibodies that compete with IgE and IL-10-producing cells, particularly T regulatory (Treg) cells, capable of suppressing established Th2 proallergic responses (7, 8). Therefore, targeting allergens to dendritic cells (DCs) - the most potent antigen-presenting cells - represents a rational approach to improve the performance of current AIT preparations (9, 10). Furthermore, due to safety concerns, hypoallergenic preparations are also desirable (10, 11).

Polymerized allergoids conjugated with nonoxidized mannan represent innovative hypoallergenic vaccines targeting DCs (12). Mannan interacts with specific C-type lectin receptors on DCs, allowing for faster and higher uptake of allergen conjugates by these cells (13, 14). Moreover, mannan-allergoid conjugates imprint DCs - or monocyte-derived DCs - with tolerogenic features that favor the induction of IL-10-producing Treg cells (15, 16).

Recently, placebo-controlled dose-finding studies have been performed with mannan allergoids derived from mites and birch pollen (17, 18). Here, we present the results of the first-in-human study, designed to evaluate the safety and efficacy of escalating doses of mannan-conjugated grass pollen allergoids (*Phleum pratense* and *Dactylis glomerata*). Both the subcutaneous and sublingual routes were evaluated in a double-dummy study design with placebo controls.

## 2 Methods

### 2.1 Trial design and ethics

**Figure 1:**
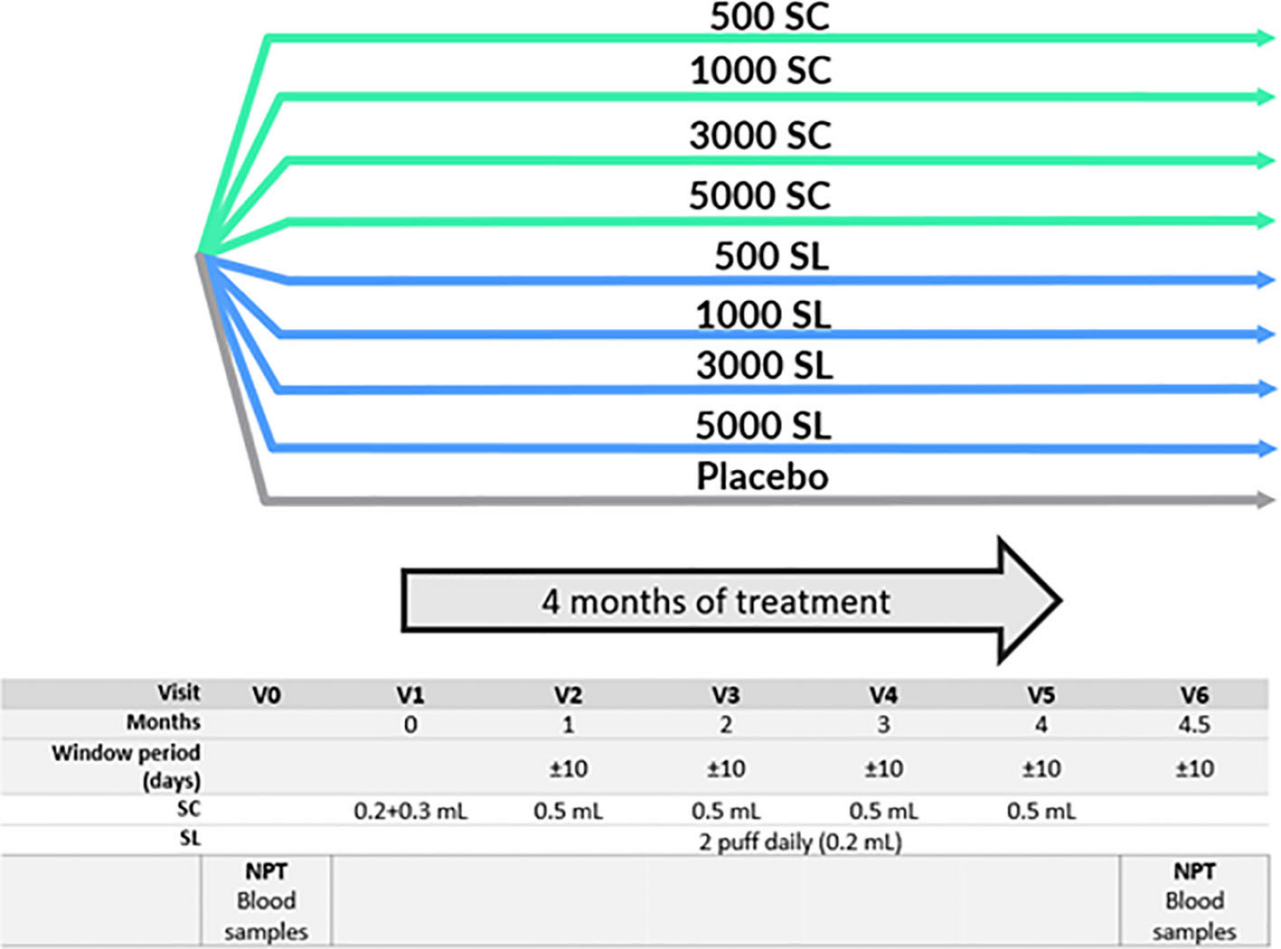
Study schedule and groups. NPT, Nasal Provocation Test. SC, Subcutaneous. SL, Sublingual. 500 SC, 1000 SC, 3000 SC and 5000 SC groups, received placebo sublingual and active subcutaneous treatment at 500, 1000, 3000 and 5000 mTU/mL respectively. 500 SL, 1000 SL, 3000 SL and 5000 SL groups, received placebo subcutaneous and active sublingual treatment at 500, 1000, 3000 and 5000 mTU/mL respectively.

The clinical trial, a phase II prospective, randomized, double-blind, placebo-controlled, double-dummy study with nine arms, was conducted in 12 centers of Spain. Most subjects were from Madrid, where allergy to grass pollen is highly relevant and some other large cities with high prevalence. Its aim was to determine the safest and most efficacious dose for either the subcutaneous (SC) or sublingual (SL) routes. **Figure 1** shows the distribution of subject groups and the trial scheme.

The study was conducted in accordance with the ICH Guideline on Good Clinical Practice (19) and the Declaration of Helsinki (20). It was approved by Ethics Committee of the Autonomous Community of Madrid and the Spanish Regulatory Authorities (AEMPS). All patients provided written informed consent. The trial was registered in EudraCT (2014–005471–88) and in ClinicalTrials.gov (NCT02654223).

### 2.2 Treatments and schedules

The investigational medicinal product was a mixture of 50% *Phleum pratense* and 50% *Dactylis glomerata* polymerized allergoids conjugated with mannan (Inmunotek, Alcalá de Henares, Spain) produced as previously described (21). Briefly, the active substances were enriched in their allergenic fraction, polymerized, and conjugated with non-oxidized mannan from *Saccharomyces cerevisiae* using glutaraldehyde. Conjugation with mannan was confirmed by nuclear magnetic resonance and the presence of Phl p 5 and Dac g 5 by mass spectrometry. The administered concentrations were 500, 1,000, 3,000 and 5,000 mTU (mannan-conjugated Therapeutic Units)/mL, corresponding respectively to 0.7, 1.4, 4.2 and 7.0 µg/mL of grass Group 5 allergen from the corresponding native extracts. The excipients in the active SC treatment were human serum albumin, sodium chloride, phenol and water for injection, while in the SL treatment they were glycerol, sodium chloride, artificial pineapple essence and water for injection. The placebo preparations for both SC and SL contained a solution identical in composition and presentation to the corresponding test products but without the active substance.

The treatment period lasted four months and occurred outside the grass pollen season (from July to April). The schedule of SC administration was five doses of 0.5 mL over four months, with the first dose divided into 0.2 and 0.3 mL, administered 30 minutes apart in alternate arms. The SL administration consisted of a daily, pump-metered dose of 0.1 mL per puff, sprayed twice under the tongue. The first dose was administered under supervision at the trial site to ensure correct administration, and subsequent doses were self-administered at home. To assess the compliance, the volume of the medication remaining in the bottles was measured at the end of the trial.

### 2.3 Sample size and subject population

Sample size was calculated based on the assumption that 60% of subjects of each group receiving active treatment and 15% of subjects in the placebo group will experience improvement. Assuming an alpha error of 0.05 and a power of 0.80, the number of subjects was 17 per group. Assuming dropouts, subjects were allocated in blocks of 20 patients, with 9 different treatments, using a list generated by the package Random software (Random Software Ltd).

**Figure 2:**
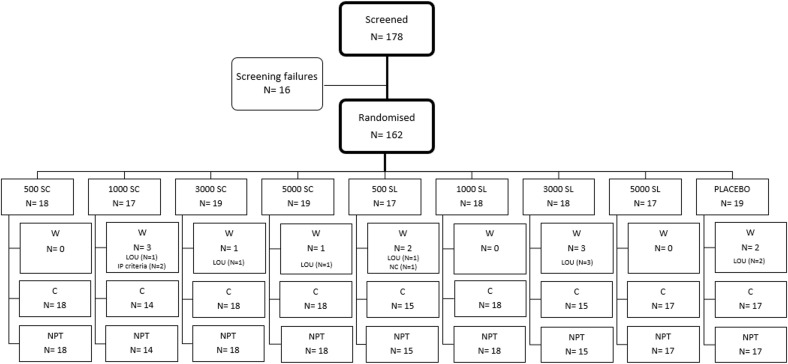
Consort diagram of the study population. C, Completed; LOU, Loss of follow-up; NPT, Nasal provocation test; NC, Non-compliant; W, Withdrawn. N, number of subjects.

Subjects screened for eligibility to enrol into the study were 178. From these, 162 were randomized, 84 males (52%) and 78 females (48%), and received, at least, one dose of treatment (intention to treat population -ITT-). Of these, 150 were evaluable for nasal provocation test (NPT) at baseline and at the end of the study (per protocol population -PP-). The CONSORT flow diagram is shown in **Figure 2**.

The study population included adolescents and adults from 14 to 58 years of age, median value (interquartile range) of 33 (26–41). All subjects had grass pollen driven allergic rhinitis/rhinoconjunctivitis, as well as positive skin prick test (wheal major diameter ≥ 6 mm) to a mixture of 6 grass pollen allergen extracts (*Phleum pratense, Dactylis glomerata, Lolium perenne, Poa pratensis, Holcus lanatus* and *Festuca elatior*) at 50 HEP/mL (Inmunotek, Spain), and at least a specific IgE class 3 to *P. pratense.* Subjects sensitized to other allergens (perennial or pollens) were allowed to be included as long as these sensitizations were considered clinically nonsignificant and, in the case of sensitizations to other pollens, the corresponding pollen season did not overlap with the NPT. The demographic characteristics of participants are shown in **Table 1**. Their pattern of allergen sensitization is shown in **Supplementary Table IS** (**Online Supplementary Information**).

**Table 1:** Demographics of study population.

		Overall	Subcutaneous (mTU/mL)				Sublingual (mTU/mL)				Placebo
			500	1000	3000	5000	500	1000	3000	5000	
		(n=162)	(n=18)	(n=17)	(n=19)	(n=19)	(n= 17)	(n=18)	(n=18)	(n=17)	(n= 19)
Sensitization status											
Grass monosensitized	n (%)	37 (22.8)	3 (16.7)	5 (29.4)	5 (26.3)	6 (31.6)	2 (11.8)	4 (22.2)	4 (22.2)	2 (11.8)	6 (31.6)
Other sensitizations	n (%)	125 (77.2)	15 (83.3)	12 (70.6)	14 (73.7)	13 (68.4)	15 (88.2)	14 (77.8)	14 (77.8)	15 (88.2)	13 (68.4)
	p-value^1^	0.762									
Gender											
Woman	n (%)	78 (48.1)	9 (50.0)	6 (35.3)	8 (42.1)	9 (47.4)	7 (41.2)	7 (38.9)	8 (44.4)	14 (82.4)	10 (52.6)
Man	n (%)	84 (51.9)	9 (50.0)	11 (64.7)	11 (57.9)	10 (52.6)	10 (58.8)	11 (61.1)	10 (55.6)	3 (17.6)	9 (47.4)
	p-value^1^	0.225									
Age (years)											
	Median (Q1, Q3)	33.0 (26.0, 41.0)	29.0 (25.0, 36.0)	36.0 (28.0, 39.0)	32.0 (26.0, 41.0)	36.0 (27.0, 44.0)	32.0 (26.0, 38.0)	39.5 (29.0, 45.0)	32.0 (22.0, 37.0)	36.0 (24.0, 41.0)	31.0 (20.0, 43.0)
	Min, Max	14, 58	14, 58	19, 52	22, 50	22, 50	17, 54	23, 56	17, 53	14, 53	15, 53
	p-value^3^	0.542									
Race											
Caucasian	n (%)	152 (93.8)	17 (94.4)	14 (82.4)	18 (94.7)	17 (89.5)	15 (88.2)	18 (100.0)	17 (94.4)	17 (100.0)	19 (100.0)
North African	n (%)	1 (0.6)					1 (5.9)				
South American	n (%)	9 (5.6)	1 (5.6)	3 (17.6)	1 (5.3)	2 (10.5)	1 (5.9)		1 (5.6)		
	p-value^2^	0.343									

^1^p: Chi-square test; ^2^p: Fisher exact test; ^3^p: Kruskal Wallis test; Values represent n (%).

### 2.4 Outcome measures

#### Primary outcome

The primary endpoint was defined as the change in pre- and post-treatment threshold concentration required to elicit a positive NPT (22, 23), being performed according to standard procedures (24) and the guidelines of the Spanish Society of Allergy and Clinical Immunology (25) in an asymptomatic phase of the patient’s disease at baseline (V0) and at the end (V6) of the study. In each city were the study was conducted, one trained healthcare professional, with the portable acoustic rhinometer (Optomic) was the responsible of conducting NPT. Thus, all subjects were challenged (at baseline and at the end) by the same person, the same rhinometer and the same methodology. NPT preparations included a negative control (saline solution) and three concentrations (0.3, 1.0 and 3.0 HEP/mL) of native non-polymerized *P. pratense* pollen extract (Inmunotek). NPT was evaluated by acoustic rhinometry (Optomic, Madrid, Spain). Briefly, after tempering the different test solutions, nasal challenges commenced with a negative control followed by increasing allergen concentrations until a positive response was elicited. The test was considered positive when the nasal cavity volume between 2 cm and 6 cm had minimum variation of 25% bilaterally together with a minimum increase ≥ 3 points in the Lebel score, or a strong variation (>40%), or an increase of ≥ 5 points in the Lebel score (26). A subject was considered to have improved if the allergen concentration required to elicit a positive nasal test at the end of the study was at least one concentration higher, i.e., three times the concentration scored at baseline.

NPT was not performed if any of the following were present: i) presence of signs and/or symptoms of allergic, viral, or infectious rhinitis in the 2 weeks prior to testing, ii) use of medications that could affect test parameters (oral or topical antihistamines, steroids, or antidepressants with antiallergic properties), iii) positive reaction with negative control.

#### Secondary outcome

Immunogenicity assessments were used as secondary endpoints. Antibody and cellular parameters were measured in blood samples collected at baseline (V0) and at the end of the study (V6).

Antibodies measured in serum included total IgE and specific IgE for *Phleum pratense* (Immulite^®^ 2000 XPi, Siemens, Germany); specific IgG4 for *Phleum pratense* (UniCAP^®^ 250, Thermo Fisher, Spain). In addition, anti-*S. cerevisiae* antibodies (ASCAs) IgG and IgA (Alegria^®^ Orgentec, Palex Medical, Spain) were tested.

Cellular parameters included the determination of Treg cells and *Phleum*-specific IL-10-secreting cells. Peripheral blood mononuclear cells (PBMC) were isolated and maintained in liquid nitrogen until use. For Treg cell analysis, PBMC were first subjected to surface staining with anti-human CD4-PerCP, CD127-PE, and CD25-APC (Miltenyi Biotec). After fixation and permeabilization, cells were stained with anti-human FOXP3–Alexa Fluor 488 (BioLegend). Flow cytometric analysis was performed using a FACSCalibur cytometer (Becton Dickinson) and Weasel v2.5 software. *Phleum*-specific IL-10-secreting cells were detected as follows. Allergen-specific cells derived from PBMC were expanded *in vitro* in the presence of IL-2 after stimulation with a pool of 20 peptides encompassing known CD4 T cell epitopes from timothy grass pollen, including allergens Phl p 1, Phl p 2, Phl p 3, Phl p 5 and Phl p 13, targeted by allergic patients (**Online Supplementary Information**). Subsequently, after 5 day-cultures, cells were rested for 4 hours in media and ELISPOT assays were carried as described (27). Briefly, cells were plated on 96-well PVDF plates (Millipore) coated with the relevant anti-IL-10 capture antibody (Mabtech) and incubated with the peptide pool (10 µM). Phytohaemagglutinin (1 µg/mL) and medium alone were used as positive and negative controls, respectively, and all conditions were run in triplicate. Peptide-specific IL-10-secreting cells were analyzed in an ELISPOT reader (ImmunoSpot 5.0, CTL Analyzers, US).

### 2.5 Safety

Safety was assessed throughout the study by recording all adverse events (AEs), both non-drug related and drug related (adverse reactions, ARs). The onset of ARs was considered immediate if it occurred within the first 30 minutes after drug administration, and delayed if it occurred later (28). Systemic reactions were graded according to the EAACI Position Paper (28). Local SC reactions were quantified by measuring the diameter of the induration. Immediate SC reactions with a diameter less than 5 cm and delayed reactions with a diameter of less than 10 cm were considered clinically irrelevant (29).

### 2.6 Statistical methods

Statistical analyses were performed with SAS v9.4 software (Cary, North Caroline, USA). Comparative analyses were performed for all variables with appropriate parametric and nonparametric tests.

The per-protocol population (PP-set) comprised 150 subjects who received all doses and completed the study without any major protocol deviations. The intention-to-treat (ITT-set) analysis included 162 subjects who received at least one dose of any active treatment or placebo. ITT was used for the comparative analysis of serology and for the safety assessment. Summary statistics are shown as frequency (%) for categorical data and median with corresponding interquartile range (Q1 and Q3) or mean ± standard deviation or 95% confidence interval (CI) for continuous data, according to the normal distribution analyzed by Shapiro-Wilk test. Chi-square or Fisher’s exact tests were used to analyze the number of subjects who improved in the primary outcome and Phi Coefficient was calculated to assess and to interpret the effect size (very strong, strong, moderate, weak, negligible when the value is higher than 0.70, 0.40, 0.30, 0.20 or 0 respectively) (30, 31). Non-parametric tests (Mann-Whitney U test for unpaired data, Wilcoxon test for paired data) for non-normal data and parametric tests (unpaired T test) for normal data were used for comparison statistics. Fold-change increase relative to the baseline value of each participant was performed in both serological and cellular analysis. The threshold for statistical significance was set at a *p* < 0.05.

## 3 Results

Out of the 162 subjects enrolled in the trial, 150 (92.6%) completed the four months of treatment, and had data of NPT at baseline and at the end, while 12 (7.4%) could not be tested for the second NPT because were lost to follow-up for various reasons (described in **Supplementary Table VS**). **Figure 2** provides details on the treatment received by each treatment group, the number of patients assigned to each group, and the reasons for discontinuation.

### 3.1 Primary endpoint: changes in nasal provocation test results

**Table 2**, **Figure 3** and **Supplementary Figure 1S** show the results of the number and percentage of subjects who experienced improvement in the NPT for each group, according to treatment (dose and route of administration) compared to the baseline test. As shown, all active groups, whether administered subcutaneously or sublingually, showed better improvements in NPT scores than the placebo group. Notably, the subjects of groups receiving the higher concentrations of 3,000 mTU/mL (3000 SC: 50% of subjects, p = 0.015; 3000 SL: 46.7%, p = 0.049) and 5,000 mTU/mL (5000 SC: 50%, p = 0.015; 5000 SL: 52.9%, p = 0.010) demonstrated a significant improvement in NPT compared to those receiving a placebo. As indicated in the same table, the effect size for the subjects with higher concentrations were categorized as “Strong” for 3,000 mTU/mL (3000 SC) and 5,000 mTU/mL (5000 SC and 5000 SL), supporting the substantial improvement in these groups (30, 31).

**Table 2:** Primary outcome results: Titrated Nasal Provocation Test.

Group	N†	Subjects with improvement	Subjects with no improvement	% of subjects with improvement	p-Value	Effect size^1^	Interpretation effect size
Placebo	17	2	15	11.8%			
500SC	18	6	12	33.3%	0.228**	-0.2567	Weak
1000SC	14	5	9	35.7%	0.198**	-0.2851	Weak
3000SC	18	9	9	50.0%	0.015*	-0.4116	Strong
5000SC	18	9	9	50.0%	0.015*	-0.4116	Strong
500SL	15	5	10	33.3%	0.209**	-0.2604	Weak
1000SL	18	6	12	33.3%	0.228**	-0.2567	Weak
3000SL	15	7	8	46.7%	0.049**	-0.3874	Moderate
5000SL	17	9	8	52.9%	0.010*	-0.4401	Strong

*Chi-square test or **Fisher`s test of the corresponding group versus placebo.

N†: N= 150 (complete, without missing data).

^1^: Phi coefficient.

**Figure 3:**
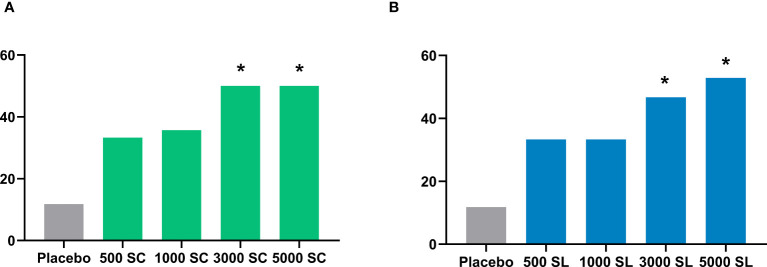
Percentage of subjects experiencing improvement in the nasal provocation test in each group administered subcutaneously **(A)** or sublingually **(B)**. Comparison with placebo, Chi-square test: *p<0.05.

### 3.2 Secondary endpoints: changes in immunological parameters

**Figure 4:**
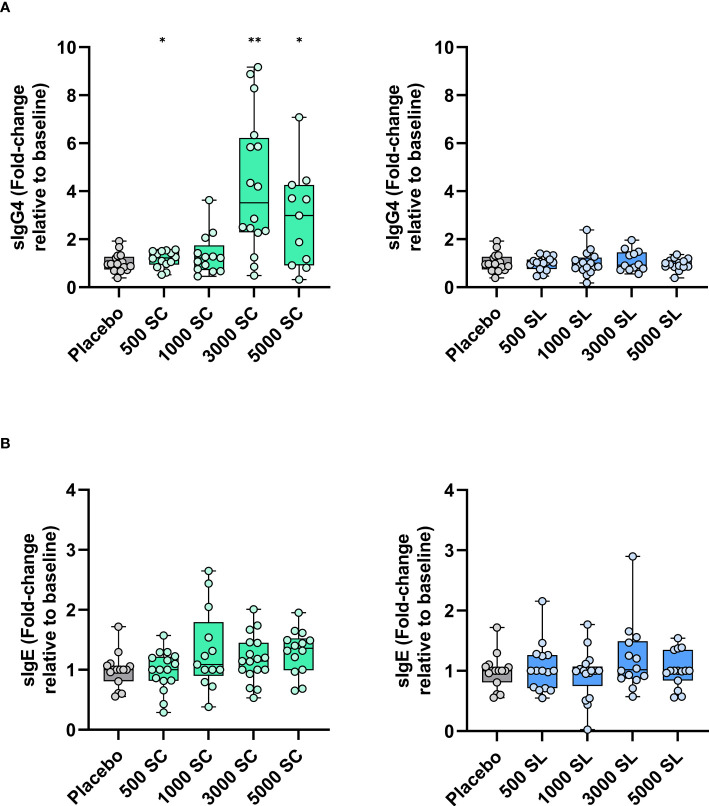
Variation (fold-change) in serum levels of grass allergen-specific antibodies [**(A)** sIgG4, **(B)** sIgE] after four months of treatment depending on dose and route of administration (subcutaneous: left panels; sublingual: right panels). Results are expressed as fold-change (median; interquartile range) relative to baseline. Comparison with placebo (Mann-Whitney test; * p< 0.05, ** p< 0.01).

**Figure 4** shows the changes in serum IgG4 and IgE antibody levels to grass pollen (*Phleum pratense*) after four months of treatment. As shown, a very significant increase from baseline was observed for IgG4 at the higher doses (3,000 or 5,000 mTU/mL) when administered subcutaneously. This increase was also significant as compared to the placebo group (**Supplementary Table IIS**, **Online Supplementary Information**). Conversely, serum specific IgG4 remained at baseline levels in all sublingual groups. Specific IgE also remained without significant variation in all groups, except for a slight but significant increase from baseline, but not from placebo (**Supplementary Table IIIS**, **Online Supplementary Information**), in the 5000 SC group (**Figure 4B**). Thus, the sIgE/sIgG4 ratio decreased significantly from baseline in the 3000 SC and 5000 SC groups (**Supplementary Table IVS**, **Online Supplementary Information**).

**Figure 5:**
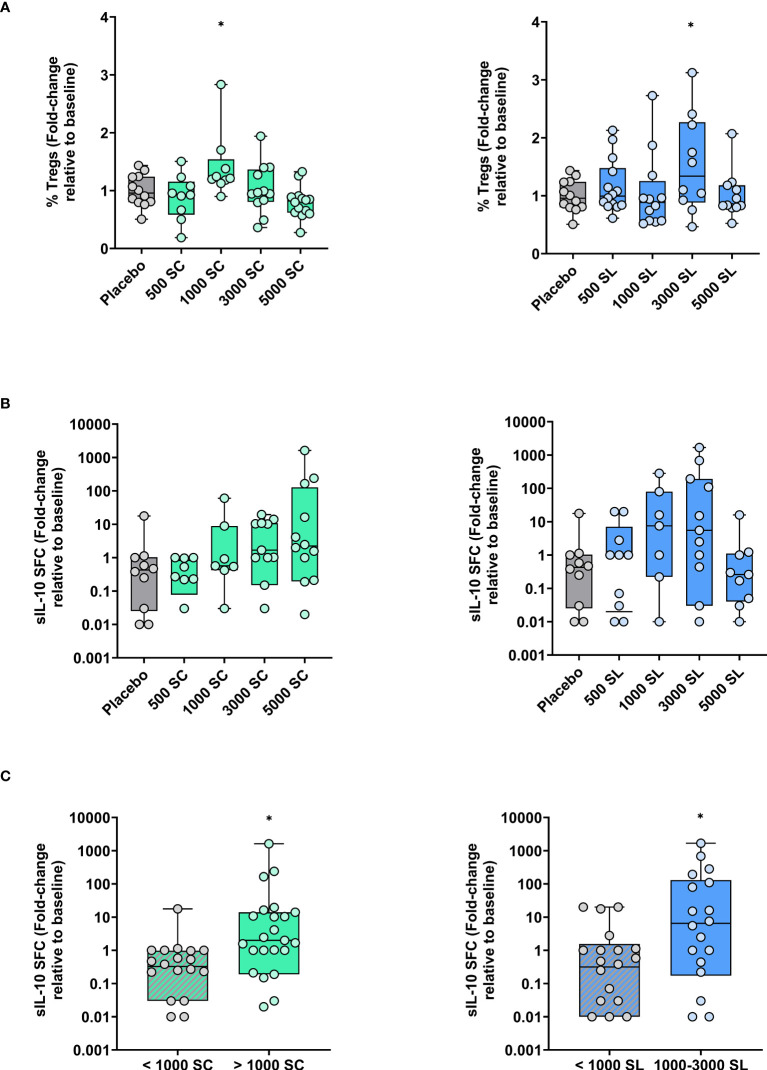
Variations (fold-change) in circulating Treg cells and grass allergen-specific IL-10-producing cells after four months of treatment depending on dose and route of administration (subcutaneous: left panels; sublingual: right panels) **(A)** Percentage of Treg cells (CD4^+^CD127^-^CD25^+^FOXP3^+^) relative to total CD4^+^ T cells. **(B)** Number of Phleum-specific spot-forming cells (SFC) secreting IL-10 after their expansion *in vitro*. **(C)** Data as in panel B but regrouping of subjects as indicated. Results are expressed as fold-change (median; interquartile range) relative to baseline. Comparison with placebo, Unpaired T test or Mann-Whitney test: * p<0.05.

Complete cell parameters were obtained from 7 to 14 subjects per group due to some samples being lost, mainly for technical reasons. Changes in circulating Treg cells and *in vitro* expanded IL-10 producing cells among available subjects are depicted in **Figure 5**. As shown, the percentage of Treg cells remained fairly stable, though a significant increase was observed in the 1000 SC group and the 3000 SL group compared to baseline or placebo (**Figure 5A**). Conversely, a clear dose-dependent trend toward an increase in IL-10-producing cells in response to *Phleum pratense* was observed in the SC group (**Figure 5B**). A similar trend was noted in the SL groups, except for the highest dose (5,000 mTU/mL), which remained unchanged. As shown in **Figure 5C**, these trends became significant when the number of subjects per group was increased by grouping those at lower doses plus placebo and those at the highest doses (excluding the 5000 SL group).

### 3.3 Safety

Overall, during the clinical trial, 22 participants (13.6% of the study population) reported a total of fifty-four adverse reactions. No Grade III or Grade IV systemic reactions were observed in any treatment group. **Table 3** shows the number of all systemic and local reactions. There was no discontinuation due to adverse events. **Supplementary Table VS** (**Online Supplementary Information**) shows the list of dropouts.

**Table 3:** Adverse reactions reported.

		Local				Systemic			
		Mild		Moderate		Grade 0		Grade 1	
		N° of events	N° of patients n (%)	N° of events	N° of patients n (%)	N° of events	N° of patients n (%)	N° of events	N° of patients n (%)
500-SC	Immediate	2	1 (5.6%)						
	Delayed					2	1 (5.6%)		
1000-SC	Immediate								
	Delayed	4	3 (17.6%)						
3000-SC	Immediate								
	Delayed	11	6 (31.6%)	3	3 (15.8%)				
5000-SC	Immediate	4	3 (15.8%)						
	Delayed	11	5 (26.3%)	9	5 (26.3%)	2	1 (5.3%)	3	2 (10.5%)
500-SL	Immediate								
	Delayed								
1000-SL	Immediate								
	Delayed								
3000-SL	Immediate	1	1 (5.6%)						
	Delayed								
5000-SL	Immediate								
	Delayed					1	1 (5.9%)	1	1 (5.9%)

Of the total fifty-four adverse reactions in the study, fifty-one were associated with active subcutaneous treatment, of which forty-four were local and seven were systemic. There were 38 delayed and 6 immediate local reactions occurring in 20 subjects. The systemic reactions were all delayed, 3 Grade 0 and 4 Grade I and occurred in a total of four subjects. On the other hand, only three adverse reactions were related to active sublingual treatment and occurred in two participants (one mild local reaction and two Grade 0 and Grade I delayed systemic reactions).

## 4 Discussion

Here, we present the results of the first-in-human study involving grass pollen polymerized allergoids conjugated with mannan. This was a multicenter, phase II, randomized, double-blind, double-dummy, placebo-controlled study conducted in Spain. The study involved patients suffering from allergic rhinitis due to grass pollen, recruited from regions where grass pollen allergy is highly prevalent (32).

The study was designed in accordance with the European Medicines Agency (33) and EAACI (34) recommendations for dose-finding studies of AIT which accept NPT as the primary outcome (33). Specific NPT allows for the inclusion of polysensitized subjects, under a controlled allergen exposure. Titrated NPT dilutions at a factor of 3 were employed due to the correlation between symptoms and the threshold for mediator release in nasal secretions during nasal challenge with grass pollen allergens (22, 23). Acoustic rhinometry was chosen to assess nasal patency because of its standardization, ease of performance, speed and minimal need for patient cooperation together with the subjective measurement of the Lebel score (25, 26). Moreover, acoustic rhinometry provides an objective and reproducible measure of nasal congestion, one of the most challenging symptoms to ameliorate in allergic rhinitis (35).

In this trial we used the same concentrations used for the dose-finding with mannan-conjugated allergoid of mites (17). The results indicated that the maximum effect was achieved with either 3,000 or 5,000 mTU/mL, irrespective of the route of administration (SC or SL). However, for any given concentration, the cumulative dose administered over the entire treatment period was almost 10 times higher via the sublingual route due to its daily administration schedule. Regarding the efficacy of both routes of administration, comparable results were observed in the primary endpoint, with approximately half of the subjects in each route requiring at least a 3-fold increase in allergen concentration to achieve a positive NPT after four months of treatment. However, the degree of clinical efficacy of each route of administration may change if other outcomes, such as symptom and/or medication scores, are considered, as noted in a previous study (17).

Immunogenicity studies indicated that both the 3,000 and 5,000 mTU/mL doses led to a significant increase in serum levels of allergen specific IgG4 when administered subcutaneously. This increase aligns with findings from previous studies on mannan-allergoid conjugates from mites or birch pollen (17, 18). Moreover, the ability of IgG induced by grass or birch pollen allergoids conjugated with mannan to compete with specific IgE has been confirmed in both preclinical (13) and clinical settings (18). In this sense, it is noteworthy that the sublingual administration route showed no effect on the increase in IgG4, or IgG (data not shown), antibodies at any tested dose, despite the positive results seen in NPT. Such a dissociation between meaningful clinical outcome and lack of serum IgG4 antibody response has also been observed with house dust mites mannan-allergoids (17). Although serum IgG antibody levels alone do not directly correlate with the clinical benefit of AIT (36), they are often associated with favorable outcomes and are considered an indicator of immunogenicity (37). However, it is recognized that the systemic IgG response is lower in SLIT than in SCIT, which in turn induces a higher IgA response both systemically and locally at the mucosal level (38).

A trend toward a dose-dependent increase in allergen-specific IL-10-producing cells was observed in the SC groups, which was statistically significant when pooling and comparing the higher and lower dose groups. A similar and significant trend was observed in the SL groups, with the notable exception of the highest dose group. Overall, these results suggest the presence of circulating allergen-specific regulatory cells after four months of administration of mannan-allergoid grass conjugates by either route. Interestingly, circulating Treg cells, while generally stable in most groups, were increased in some SC or SL groups where IL-10-producing cells were also observed. Nevertheless, a direct relationship between IL-10-producing and Treg cells was not seen. Although this may not be surprising given the different readouts (allergen-specific versus non-specific), and the fact that the allergen-specific IL-10-producing cells were expanded *in vitro* prior to quantification, other IL-10-producing regulatory cells, like Breg (39) or Tr1 (40) cells, cannot be ruled out in absence of phenotypic characterization. In this sense, the use of a set of peptides specifically selected/designed for CD4^+^ T cell activation (**Supplementary Table VIS**, **Online Supplementary Information**) does not support the former possibility. Regardless of the cellular source, these results are noteworthy because AIT-induced IL-10 production is key to downregulating allergen-specific Th2 and associated inflammatory responses (7, 8).

No significant safety concerns were identified, in line with previous clinical trials with mannan-allergoid conjugates derived from dust mites and birch pollen (17, 18). No Grade III or IV systemic reactions were reported in any study group, while Grade I or II reactions were mostly delayed type. Most local reactions in the subcutaneous group were mild, typically occurring after the first injections, and none led to withdrawal from the study. In the active sublingual groups, adverse reactions were exceptionally low, with only two mild systemic reactions (Grade 0 and Grade I) and a single mild immediate local reaction reported. On the other hand, the antibody response to *S. cerevisiae* mannan was negligible. No significant changes in serum IgG-ASCA or IgA-ASCA levels were observed (**Supplementary Tables VIIS**–**VIIIS**, **Online Supplementary Information**), consistent with a previous clinical study with dust mites allergoids conjugated with mannan (17).

In conclusion, this study indicates that either 3,000 mTU/mL or 5,000 mTU/mL of grass pollen polymerized allergoids conjugated with mannan met the primary efficacy endpoint with an excellent safety profile by both the subcutaneous and the sublingual routes. This should be confirmed in subsequent Phase III clinical trials (18).

## Data availability statement

The data that support the findings of this study are available from the corresponding author upon reasonable request.

## Ethics statement

The studies involving humans were approved by Ethics Committee of the Autonomous Community of Madrid and the Spanish Regulatory Authorities (AEMPS). All patients provided written informed consent. The trial was registered in EudraCT (2014–005471–88) and in ClinicalTrials.gov (NCT02654223). The studies were conducted in accordance with the local legislation and institutional requirements. Written informed consent for participation in this study was provided by the participants’ legal guardians/next of kin.

## Author contributions

PO: Investigation, Project administration, Supervision, Writing – review & editing. MB: Investigation, Writing – review & editing. JS: Investigation, Writing – review & editing. AM: Investigation, Writing – review & editing. IO: Investigation, Writing – review & editing. ES: Investigation, Writing – review & editing. AAl: Investigation, Writing – review & editing. RC: Conceptualization, Formal analysis, Funding acquisition, Methodology, Project administration, Resources, Supervision, Validation, Writing – review & editing. SP: Data curation, Writing – original draft, Writing – review & editing. MG-P: Writing – review & editing, Investigation. JS-T: Investigation, Writing – review & editing. CB-V: Investigation, Writing – review & editing. AAn: Investigation, Writing – review & editing. IS: Investigation, Writing – review & editing. PR: Investigation, Writing – review & editing. OP: Writing – review & editing, Investigation. JLS: Conceptualization, Formal analysis, Funding acquisition, Methodology, Project administration, Resources, Writing – original draft, Writing – review & editing. MC: Conceptualization, Data curation, Formal analysis, Funding acquisition, Methodology, Project administration, Resources, Software, Supervision, Validation, Visualization, Writing – original draft, Writing – review & editing.
