# Supplementary material for: Grass pollen allergoids conjugated with mannan for subcutaneous and sublingual immunotherapy: a dose-finding study
Source: Front Immunol. 2024 Jun 26;15:1431351. doi: 10.3389/fimmu.2024.1431351 (PMC11233432; doi:10.3389/fimmu.2024.1431351)
Supplement: Supplementary file 1 [file DataSheet_1.pdf]

Content

1. Other allergen sensitisations of the subjects 2
2. Nasal Provocation Test 3
3. Allergen specific antibodies (serology) 4
   3.1. Phleum specific IgG4 4
   3.2. Phleum specific IgE 4
   3.3 Ratio sIgE/IgG4 5
4. Drop-outs 5
5. Cell analysis 6
   5.1. Phleum specific CD4 T cell epitopes 6
   5.2. Determination of Phleum-specific IL-10 producing cells 7
6. Safety 8
6.1. Adverse Events (AEs) 9
6.2. ASCA 10
   6.2.1. IgG 10
   6.2.2. IgA 10
7. References 11

1. Other allergen sensitisations of the subjects

Table IS. Other allergen sensitizations of subject, skin prick test results.

Allergen			Subcutaneous				Sublingual				
		Overall (n=162)	500 (n=18)	1000 (n=17)	3000 (n=19)	5000 (n=19)	500 (n=17)	1000 (n=18)	3000 (n=18)	5000 (n=17)	Placebo (n=19)
Tree pollen	n (%)	110 (67.9)	14 (77.8)	12 (70.6)	10 (52.6)	12 (63.2)	12 (70.6)	12 (66.7)	13 (72.2)	15 (88.2)	10 (52.6)
	p-value[1]	0.380									
Dander	n (%)	39 (24.1)	8 (44.4)	3 (17.6)	4 (21.1)	3 (15.8)	3 (17.6)	5 (27.8)	4 (22.2)	5 (29.4)	4 (21.1)
	p-value[1]	0.626									
Weed pollen	n (%)	37 (22.8)	3 (16.7)	1 (5.9)	6 (31.6)	5 (26.3)	2 (11.8)	6 (33.3)	6 (33.3)	5 (29.4)	3 (15.8)
	p-value[1]	0.386									
Mites	n (%)	21 (13.0)	2 (11.1)	1 (5.9)	1 (5.3)	3 (15.8)	2 (11.8)	1 (5.6)	3 (16.7)	2 (11.8)	6 (31.6)
	p-value[1]	0.093									
Molds	n (%)	7 (4.3)	1 (5.6)			1 (5.3)	1 (5.9)		2 (11.1)	1 (5.9)	1 (5.3)
	p-value[1]	0.779									
Others	n (%)	8 (4.9)	1 (5.6)	1 (5.9)	1 (5.3)		2 (11.8)		1 (5.6)	1 (5.9)	1 (5.3)
	p-value[1]	0.882									

[1] p: Chi-square test

2. Nasal Provocation Test

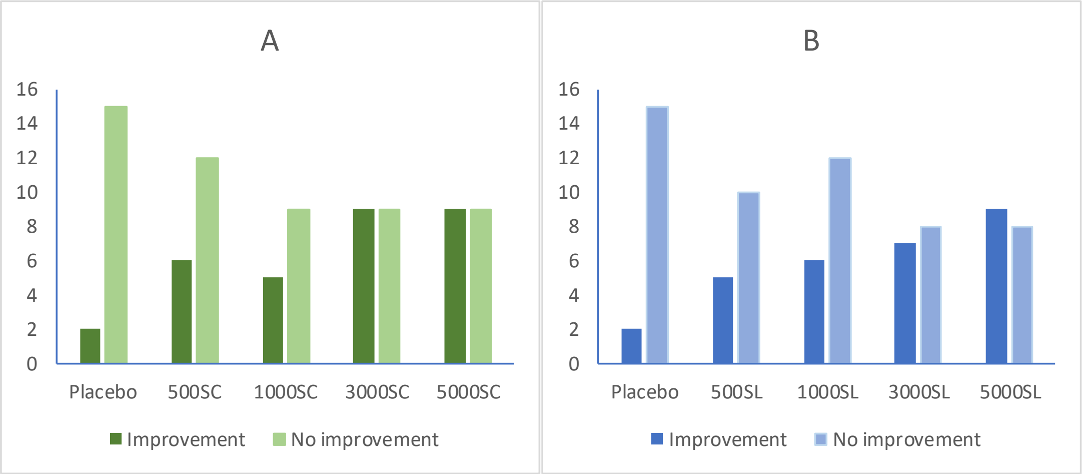

Figure 1S. Number of subjects experiencing or not improvement in the nasal provocation test in each group administered subcutaneously (Panel A) or sublingually (Panel B).

3. Allergen specific antibodies (serology)

3.1. Phleum specific IgG4

Table IIS. Median (Q1 and Q3) of Phleum Specific IgG4 values and comparative statistics

***P. pratense* specific IgG4 (mg/L)**

Group	Baseline Median (Q1, Q3)	Final Median (Q1, Q3)	Foldchange from baseline	p*	p**
Placebo	0.5 (0.2, 0.9)	0.5 (0.2, 1.2)	0.97 (0.75, 1.22)	0.686	
500 SC	0.5 (0.1, 0.6)	0.6 (0.2, 0.8)	1.38 (1.05, 1.54)	0.021	0.043
1000 SC	0.3 (0.2, 0.5)	0.7 (0.2, 1.0)	1.24 (0.75, 2.06)	0.051	0.251
3000 SC	0.9 (0.3, 1.4)	2.7 (2.2, 5.4)	4.19 (2.34, 6.34)	<.001	<.001
5000 SC	0.4 (0.2, 1.1)	1.3 (0.2, 2.5)	3.66 (1.17, 4.44)	0.007	0.011
500 SL	0.6 (0.3, 0.8)	0.5 (0.2, 0.7)	1.03 (0.75, 1.15)	0.8911	0.667
1000 SL	0.7 (0.3, 1.2)	0.6 (0.4, 0.8)	0.91 (0.79, 1.27)	0.369	0.973
3000 SL	0.3 (0.2, 0.6)	0.3 (0.1, 1.0)	1.14 (0.78, 1.60)	0.636	0.353
5000 SL	0.4 (0.3, 0.6)	0.4 (0.3, 0.6)	0.98 (0.85, 1.15)	0.590	0.830

p*: Wilcoxon test (comparison baseline - final)
p**: Mann-Whitney test (comparison vs placebo)

3.2. Phleum specific IgE

Table IIIS. Median (Q1 and Q3) of Phleum Specific IgE values and comparative statistics

***P. pratense* specific IgE (KU/L)**

Group	Baseline Median (Q1, Q3)	Final Median (Q1, Q3)	Foldchange from baseline	p*	p**
Placebo	77.1 (28.5, 100.0)	65.0 (33.1, 100.0)	1.00 (0.96, 1.11)	0.569	
500 SC	67.0 (25.4, 91.8)	54.7 (32.4, 93.4)	1.00 (0.82, 1.23)	0.358	0.877
1000 SC	55.8 (28.7, 96.0)	100 (38.3, 100.0)	1.16 (1.00, 2.05)	0.147	0.255
3000 SC	75.9 (53.5, 100.0)	97.4 (43.4, 100.0)	1.15 (1.00, 1.45)	0.241	0.402
5000 SC	37.7 (16.8, 67.2)	68.3 (27.7, 100.0)	1.40 (1.00, 1.62)	0.003	0.082
500 SL	69.4 (28.5, 100.0)	61.1 (35.6, 100.0)	1.00 (0.71, 1.26)	0.577	0.497
1000 SL	62.9 (44.2, 100.0)	67.1 (38.3, 100.0)	1.00 (0.95, 1.03)	0.519	0.371
3000 SL	41.2 (23.7, 77.2)	58.0 (23.2, 100.0)	1.02 (0.87, 1.47)	0.424	1.000
5000 SL	62.9 (28.1, 100.0)	60.7 (26.5, 100.0)	1.00 (0.97, 1.37)	0.569	0.904

p*: Wilcoxon test (comparison baseline - final)
p**: Mann-Whitney test (comparison vs placebo)

3.3 Ratio sIgE/IgG4

Table IVS. Median (Q1 and Q3) of Ratio sIgE/IgG4 values and comparative statistics

***P. pratense* specific IgE/IgG4 (KU/L)**			
**Group**	**IgE/IgG4 baseline**	**IgE/IgG4 Final**	**p***
Placebo	163.1 (33.8, 227.9)	100.0 (50.4, 190.0)	0.284
500 SC	94.3 (45.0, 178.6)	102.9 (60.2, 180.0)	0.109
1000 SC	186.6 (93.5, 289.3)	144.9 (68.8, 526.3)	0.903
3000 SC	89.7 (40.2, 223.8)	35.0 (18.7, 46.3)	0.001
5000 SC	65.4 (49.6, 138.9)	45.2 (26.7, 94.4)	0.048
500 SL	85.6 (77.2, 168.0)	99.7 (77.8, 181.8)	0.720
1000 SL	84.7 (56.8, 142.6)	98.0 (49.1, 166.7)	0.548
3000 SL	169.5 (59.4, 358.8)	195.0 (70.0, 312.5)	0.761
5000 SL	132.9 (53.2, 252.5)	140.4 (53.0, 195.8)	0.679

p*: Wilcoxon test (comparison baseline - final)

4. Drop-outs

The following table shows the list of abandons and the cause of these abandons.

Table VS. Drop-outs

Group	Last study visit	Premature termination reasons
Placebo	4	Loss to follow-up
Placebo	3	Loss to follow-up
1000 SC	4	Loss to follow-up
1000 SC	1	Investigator's criteria: inclusion criteria not met: specific IgE < 3 kU/L
1000 SC	3	Investigator's criteria: recurrence of asthma symptoms
3000 SC	5	Loss to follow-up
5000 SC	5	Loss to follow-up
500 SL	2	Loss to follow-up
500 SL	3	Discontinuation > 15 days of SL treatment
3000 SL	4	Loss to follow-up
3000 SL	3	Loss to follow-up
3000 SL	4	Loss to follow-up

5. Cell analysis

5.1. Phleum specific CD4 T cell epitopes

Experimentally verified timothy grass specific CD4 T cell epitopes targeted by allergic subjects were retrieved from the Immune Epitope Database (IEDB) [PMID: 22610854] after the following search: 1) Epitope, linear peptide; 2) Organism, *Phleum pratense*; 3) Host, human; 4) T cell assay with positive result, 5) Class II restriction and 6) Disease Allergy. CD-HIT [PMID: 16731699] was used to identify and cluster CD4 T cell epitopes with overlapping amino acid sequences (100 % identity threshold) [(1)]. Clusters were processed and CD4 T cell epitopes with ≥ 7 residue-overlaps were combined into extended peptides as described in [(2)]. As a result, 20 peptides bearing one or more CD4 T cell epitopes were selected for peptide synthesis (Table VIS). Peptides were synthesized by ProteoGenix (Schiltigheim, France) at ≥95% purity as confirmed by reversed-phase high-performance liquid chromatography (RP-HPLC). Lyophilized peptides were dissolved in 40% dimethyl sulfoxide, diluted in ultra-pure water to a peptide concentration of 5 mM, and stored at −80°C until use.

Table VIS. Peptide sequences contained in Phleum peptide pool

**Peptide**	**ACC** [1]	**Position**	**Protein**	**IEDB** [2]	**HLA II restriction** [3]
STWYGKPTGAGPKDN	P43213	46-60	Phl p1	127151	HLA-DQB1*03:01, HLA-DR
KPPFSGMTGCGNTPI	P43213	71-85	Phl p1	126461	HLA-DR
FEIKCTKPEACSGEPVVVHI	P43213	96-115	Phl p1	125972, 15558, 30090, 127213, 32685	HLA-DQ, HLA-DR,
SGIAFGSMAKKGDEQ	Q40967	131-145	Phl p1	127062	HLA-DQB1*03:01, HLA-DQB1*03:02, HLA-DR
GELELQFRRVKCKYP	P43213	151-165	Phl p1	126096, 35586	HLA-DRB1*11:01, HLA-DR
TFHVEKGSNPNYLALLVKYVN GDGD	P43213	171-195	Phl p1	127200, 126404, 48726, 126799, 34855	HLA-DQB1*03:01, HLA-DQB1*03:02, HLA-DRB1*11:01, HLA-DRB1*04:04
EHGSDEWVAMTKGEGGVW TF	P43214	61-80	Phl p2	125889, 125952	HLA-DRB1*03:01, HLA-DRB1*11:01
LQGPFNFRFLTEKGMKNVFD DVVPEKYTIG	P43214	86-115	Phl p2	126609, 126750, 127192, 126458	HLA-DQB1*02:01, HLA-DRB5*01:01
AVQVTFTVQKGSDPKKLVLNI KYTRPGDSL	Q69B42	1-30	Phl p3	125756, 126063, 126177, 126442	HLA-DPB1*05:01, HLA-DPB1*14:01, HLA-DQB1*03:01, HLA-DRB1*03:01, HLA-DRB1*11:01, HLA-DRB1*13:01, DRB3*01:01
EEWEPLTKKGNVWEV	Q69B42	41-55	Phl p3*	125883	HLA-DRB1*08:01, HLA-DRB1*11:01
NVWEVKSSKPLVGPF	Q69B42	51-65	Phl p3*	126796	HLA-DQ, HLA-DRB1*08:01, HLA-DRB1*11:01, HLA-HLA-DRB3*02:02, DRB4*01:03
LVGPFNFRFMSKGGMRNVF DEVIPT	Q69B42	61-85	Phl p3*	126639, 126751, 127077	HLA-DQ, HLA-DRB1*11:01, HLA-DRB1*15:01

NRNNTFKPFAEYKSDYVYQPFPK	Q2I6V7	353-375	Phl p4	126784, 125956	HLA-DRB1*11:01
NAGFKAALAAAAGVPPADKY	O81341	71-90	Phl p5	126739, 125642	HLA II
PANDKFTVFEAAFNDAIK	O81341	208-225	Phl p5	177204, 125827	HLA-DPB1*05:01, HLA-DPB1*14:01, HLA-DRB1*04:01
VIPAGELQVIEKVDAAFKVA	O81341	181-200	Phl p5	127300, 176956, 125917, 39004	HLA-DPB1*14:01, HLA-DQB1*03:01, HLA-DPB1*05:01
AFKVAATAANAAPAN	O81341	196-210	Phl p5	125665	HLA II
AYESYKFIPALEAAVKQAYAATVAAA	O81341	231-256	Phl p5	125764, 177365, 227913, 74450, 177368, 126392, 177118, 864710, 125857, 863603, 125857	HLA-DQB1*03:01, HLA-DRB1*01:01, HLA-DRB1*04:01, HLA-DRB1*04:04, HLA-DRB4*01:03
ATVATAPEVKYTVFETALKKAITAMS	O81341	251-276	Phl p5	125746, 177337, 125715, 14758, 177355, 177334	HLA-DPB1*04:01, HLA-DPB1*04:02
LAKYKANWIEIMRIK	Q9XG86	96-110	Phl p13	126525	HLA-DPB1*05:01, HLA-DPB1*14:01, HLA-DRB3*02:02, HLA-DRB5*01:01

[1] Uniprot accession of allergen; [2]IEDB accession number of CD4 T cell epitopes encompassed by the peptide; [3] Experimental HLA II restriction information of CD4 T cell epitopes as recorded in IEDB. * Epitopes incorrectly annotated in IEDB as belonging to Phl p2 protein. Location of peptides in source allergens was verified using BLAST [PMID: 9254694]

5.2. Determination of Phleum-specific IL-10 producing cells

In vitro expansion of Phleum-specific T cells

Peripheral blood mononuclear cells (PBMCs) were obtained by Ficoll density gradient centrifugation (800g, 20 minutes) from patient's blood samples collected at baseline and at the end of the study and were kept in liquid nitrogen until processing. For the *in vitro* expansion of *Phleum pratense*-specific T lymphocytes, PBMCs from allergic patients were stimulated with the pool of Phleum peptides 10 µM (Proteogenix, France) and 10 U/ml of IL-2 (Immunotools, Germany). PBMCs were kept at 37 °C and 5% CO_2 for 5 days with additional doses of the stimuli on day three of the expansion. RPMI 1640 (Gibco, NY, USA) supplemented with 5% of human serum (Gibco, NY, USA), 100 U/ml penicillin, 100 µg/ml streptomycin, 2 mM L-glutamine (Lonza, Walkersville, USA) was used. The expanded cells were washed twice with PBS and allowed to rest for 4 hours in RPMI 1640 without human serum or stimulation prior to ELISPOT assay development.

ELISPOT assays

96-well PVDF ELISPOT plates (Mabtech, Sweden) were activated with 20 µl of ethanol for 1 minute, washed with PBS (Gibco) and coated with anti-IL-10 capture antibody (mAb 9D7; Mabtech, Sweden). Plates were incubated for 48 hours at 4 °C with the capture antibody, washed with PBS and blocked for 30 minutes with RPMI 1640 (Gibco, NY, USA) supplemented with 10% fetal bovine serum (FBS) (Gibco, NY, USA).

PBMCs from allergic patients were added to the ELISPOT plates and incubated for 24 hours at 37 °C and 5% CO_2. Phleum-specific IL-10 responses were detected by stimulating PBMCs

with 10 µM of the Phleum peptide pool in the ELISPOT plate. Basal cytokine production in the absence of stimuli and response to phytohemagglutinin (PHA) (1 µg/ml) (Sigma-Aldrich, Germany) were evaluated as negative and positive control for cytokine production, respectively.

SFC detection

The number of spot forming cells (SFC) was detected following manufacturer's instructions (Mabtech). In brief, plates were washed with PBS and incubated for 2 hours with anti-IL-10 detection antibody (mAb 12G8-biotin; Mabtech, Sweden). Subsequently, plates were washed with PBS and Streptavidin-ALP (1:1000) (Mabtech, Sweden) was added for 1 hour at room temperature. After several washes, 100 µl of the BCIP/NBT substrate (Mabtech, Sweden) was added. The enzymatic reaction was stopped with tap water and plates were allowed to dry for at least 48 hours before being analyzed. SFC were counted with an ELISPOT reader (ImmunoSpot 5.0, CTL Analyzers, LLC, OH, USA) considering a minimum spot size of 500 μm^2. ELISPOT assays were run in triplicate and the mean ± standard deviation of the basal control was subtracted to each value obtained.

6. Safety

All adverse events occurring during the trial were recorded and assessed. These events were thoroughly explored, both during scheduled visits and at any time in the subject reporting an abnormal incidence. EMA Good Pharmacovigilance Practice Guidelines [(3)] were followed. The adverse events could be divided in non-drug related or drug related adverse events (also known as adverse reactions).

Adverse reactions known to be related to the intrinsic (allergenic) properties of the product were classified as:

- Depending on the site of appearance:
    - Local: appear in the place of administration. The severity of these reactions was classified as:
        - Mild: event that the subject easily tolerates and that causes minimal discomfort without interfering with daily activities.
        - Moderate: event that produces discomfort in a way that interferes with activities of daily living.
        - Intense or severe: event that prevents daily activities.
    - Systemic: appear in another part of the body different from the site of administration. The severity of these adverse reactions was graded according to the following grades: [(4)]
        - Grade 0: No symptoms or nonspecific symptoms.
        - Grade 1: Mild systemic reactions: Localised urticaria, mild rhinitis that responds adequately to antihistamines or mild asthma (PEF < 20% reduction from baseline).

- Grade 2: Moderate systemic reaction. Usually slow onset (> 15 minutes) of generalised urticaria and/or moderate asthma (PEF < 40% reduction from baseline). Moderate limitation of the activity, with little medical intervention or hardly needing therapy.
- Grade 3: Severe systemic reaction. Rapid onset (< 15 minutes) of generalised urticaria, angioedema or severe asthma (PEF > 40% reduction from baseline). Severe limitation of activity that requires some type of therapy or medical assistance with possible hospitalization.
- Grade 4: Anaphylactic shock. Rapid reaction with itching, reddening, erythema, generalised urticaria, stridor (angioedema), immediate severe asthma, hypotension, etc. requiring intensive treatment. Extreme limitation of activity, necessary therapy, medical intervention required, probable hospitalization or palliative care.

- Depending on the time of appearance:
    - Immediate: those that appeared within the first 30 min after administration of the treatment.
    - Delayed: those that appeared later than the first 30 min after administration of the treatment.

The study population received the treatment for 4 months. 178 patients were screened for eligibility, of which 162 were included. In the end, 12 patients dropped and 150 completed the study.

6.1. Adverse Events (AEs)

In this study 128 events were reported, 54 were considered to be adverse drug related (related to the IMP; adverse reactions) and 74 were non-drug related adverse events (not related to IMP).

Regarding the 74 non-drug related adverse events registered in a total of 37 (22.8%) participants, 11 events were experienced in a total of 7 subjects in the placebo group. In the groups receiving subcutaneous active treatment 35 events were reported by a total of 14 subjects. On the other hand, in groups receiving sublingual active treatment, 28 non-drug related adverse events were experienced by a total of 16 participants. Of all these events, 61 were classified as mild and 13 as moderate.

In reference to the drug related adverse events (adverse reactions), 54 events were reported in a total of 22 subjects, 45 were local and 9 systemic. In the groups receiving subcutaneous active treatment 51 events were reported, of which 44 were local and 7 systemic. Of the 45 local adverse reactions, 33 were described as mild and 12 as moderate.

The most common drug related adverse events were local reactions (in the injection site), no other adverse reaction was experienced by more than 5% of the study population.

6.2. ASCA

6.2.1. IgG

Table VIIS. Median (Q1 and Q3) of IgG ASCA values (mg/L) and comparative statistics (Wilcoxon test).

Group	Baseline Median (Q1, Q3)	Final Median (Q1, Q3)	p* N (82)
Placebo	6.75 (2.10, 20.50)	7.90 (2.90, 10.00)	1.000
500 SC	3.70 (1.40, 7.10)	3.30 (1.50, 7.70)	1.000
500 SL	2.30 (1.25, 4.60)	0.70 (0.50, 1.40)	0.063
1000 SC	1.55 (0.95, 3.00)	1.65 (0.80, 2.70)	0.500
1000 SL	1.80 (1.05, 10.15)	3.00 (1.30, 11.00)	0.594
3000 SC	2.00 (0.70, 4.80)	2.90 (0.50, 5.10)	0.195
3000 SL	1.65 (0.50, 4.65)	1.80 (0.90, 2.50)	0.262
5000 SC	3.20 (1.80, 8.90)	2.35 (1.10, 6.80)	0.688
5000 SL	2.50 (1.60, 2.80)	3.70 (2.40, 8.20)	1.000

p*: Wilcoxon test (comparison baseline - final)

6.2.2. IgA

Table VIIIS. Median (Q1 and Q3) of IgA ASCA values (mg/L) and comparative statistics (Wilcoxon test).

Group	Baseline Median (Q1, Q3)	Final Median (Q1, Q3)	p*
Placebo	4.20 (2.90, 7.00)	5.10 (2.80, 7.80)	0.008
500 SC	4.60 (2.10, 9.60)	4.30 (3.00, 9.80)	0.963
1000 SC	5.20 (3.40, 7.40)	4.10 (2.60, 6.80)	0.370
3000 SC	2.80 (1.80, 4.60)	2.70 (1.70, 5.30)	0.056
5000 SC	3.60 (2.20, 6.30)	4.40 (3.00, 13.00)	0.980
500 SL	2.70 (2.00, 6.20)	2.00 (1.70, 4.60)	0.236
1000 SL	4.90 (3.80, 10.00)	3.90 (2.30, 6.00)	0.435
3000 SL	3.45 (1.70, 5.20)	2.50 (1.20, 3.60)	0.930
5000 SL	2.90 (2.50, 6.20)	2.70 (2.20, 4.80)	0.530

p*: Wilcoxon test (comparison baseline - final)

7. References

1.- Fu L, Niu B, Zhu Z, Wu S, Li W. CD-HIT: accelerated for clustering the next-generation sequencing data. Bioinformatics 2012; 28(23):3150-2.

2.- Quinzo MJ, Lafuente EM, Zuluaga P, Flower DR, Reche PA. Computational assembly of a human Cytomegalovirus vaccine upon experimental epitope legacy. BMC Bioinformatics 2019; 20(Suppl 6):476.

3.- European_Medicines_Agency. Good pharmacovigilance practices (GVP). 2014.

4.- Guidance for Industry: Toxicity Grading Scale for Healthy Adult and Adolescent Volunteers Enrolled in Preventive Vaccine Clinical Trials. In: Services USDoHaH, Administration FaD, Research CfBEa, editors.; 2007.
